# Genome-wide characterization of ovate family protein gene family associated with number of seeds per silique in *Brassica napus*

**DOI:** 10.3389/fpls.2022.962592

**Published:** 2022-09-14

**Authors:** Jie Liu, Yupo Wu, Xiaobo Cui, Xiong Zhang, Meili Xie, Lijiang Liu, Yueying Liu, Junyan Huang, Xiaohui Cheng, Shengyi Liu

**Affiliations:** Key Laboratory of Biology and Genetic Improvement of Oil Crops, Ministry of Agriculture and Rural Affairs of the PRC, Oil Crops Research Institute, Chinese Academy of Agricultural Sciences, Wuhan, China

**Keywords:** ovate family protein, *Brassica napus*, GWAS, yield traits, negative regulator, silique development

## Abstract

Ovate family proteins (OFPs) were firstly identified in tomato as proteins controlling the pear shape of the fruit. Subsequent studies have successively proved that OFPs are a class of negative regulators of plant development, and are involved in the regulation of complex traits in different plants. However, there has been no report about the functions of OFPs in rapeseed growth to date. Here, we identified the OFPs in rapeseed at the genomic level. As a result, a total of 67 members were obtained. We then analyzed the evolution from *Arabidopsis thaliana* to *Brassica napus*, illustrated their phylogenetic and syntenic relationships, and compared the gene structure and conserved domains between different copies. We also analyzed their expression patterns in rapeseed, and found significant differences in the expression of different members and in different tissues. Additionally, we performed a GWAS for the number of seeds per silique (NSPS) in a rapeseed population consisting of 204 natural accessions, and identified a new gene *BnOFP13_2* significantly associated with NSPS, which was identified as a novel function of OFPs. Haplotype analysis revealed that the accessions with haplotype 3 had a higher NSPS than other accessions, suggesting that *BnOFP13_2* is associated with NSPS. Transcript profiling during the five stages of silique development demonstrated that *BnOFP13_2* negatively regulates NSPS. These findings provide evidence for functional diversity of OFP gene family and important implications for oilseed rape breeding.

## Introduction

Oilseed crops are both an important source of edible vegetable oil and a valuable material of animal feed (Felten et al., 2013; D’Avino et al., 2015). As the second largest source of edible vegetable oil, *Brassica napus* (*B. napus*) provides about 13–16% of the total edible vegetable oil for the world (Wang et al., 2018), and the market demand for it is still increasing in recents. Therefore, great efforts have been made to improve its yield. Plant architecture, including main inflorescence length, plant height and branch number, indirectly affects the oilseed rape yield (Reinhardt and Kuhlemeier, 2002; Wang and Li, 2008; Li et al., 2019a), while siliques per plant (SPP), number of seeds per silique (NSPS), and total seed weight (TSW) directly determine the plant yield (Clarke and Simpson, 1978). Similarly, in *Arabidopsis*, the silique is closely associated with the final yield, and silique-related traits such as silique length (SL) and silique volume (SV) affect the morphology and photosynthetic substances, thus influencing the yield (Ferrándiz et al., 1999). In siliques, NSPS and SL are more important traits. In recent years, many QTLs controlling SL and NSPS have been identified at almost all chromosomes through QTL mapping and genome wide association study (GWAS) (Fu et al., 2015; Wang et al., 2016; Yang et al., 2016, 2017; Luo et al., 2017; Zhu et al., 2020). To date, two genes controlling the SL, *BnaA9.ARF18* and *BnaA9.CYP78A9*, have been cloned in *B. napus*, which affect SL by regulating cell elongation of the silique wall (Liu et al., 2015a; Shi et al., 2019). Additionally, *BnaC9.SMG7B* has been cloned as a positive regulator of NSPS, which regulates the formation of normal female gametophyte and finally determines the formation of mature ovules (Li et al., 2015). In recent studies, *BnaA08g07940D* and *BnaA08g07950D* were identified as putative candidate genes of a major QTL controlling NSPS by fine mapping (Jiao et al., 2021). Besides, in *Brassica juncea*, *BjCLV1* was found to affect NSPS through the formation of trilocular siliques (Wang et al., 2021). In *Arabidopsis*, cytokinin and brassinosteroid were found work coordinately to promote ovule initiation and then increase NSPS (Zu et al., 2022). Certainly, the yield is also affected by diseases such as Sclerotinia stem rot, clubroot, blackleg disease and stem canker. Fortunately, many resistance QTLs have been identified. Therefore, integration of these elite QTL alleles controlling different traits into elite cultivars with better plant architecture may be a promising strategy to improve the yield of oilseed rape.

Domestication of fruit-bearing crops involves long-term artificial selection from various wild plant species, and a significant hallmark in this process is the explosive increase in fruit shape variations (Williams, 1965; Hopping et al., 1986). The recessive locus ovate controlling the pear shape of fruit and elongated fruit shape in tomato was identified about one hundred years ago, but the gene was cloned until 20 years ago (Price and Drinkard, 1908; Liu et al., 2002). The ovate proteins were identified as a class of negative regulators in plant development, which contain a C-terminal conserved domain and Von Willebrand factor type C domain, which are conserved in tomato, rice and *Arabidopsis* (Liu et al., 2002; Wang et al., 2011). Subsequently, studies of plant ovate family proteins (OFPs) have been successively reported. Researchers are increasingly aware of their functions though the exact mechanism remains poorly understood. OFPs play important roles in plant growth and development, and their functions have been studied in both model plants and crops. In *Arabidopsis*, *AtOFP5* affects the cell-fate switch of synergid to egg cell in mature embryo sacs by suppressing the ectopic activity of BELL-KNOX TALE complex (Pagnussat et al., 2007); *AtOFP4* and *AtOFP1* are involved in secondary cell wall formation; and the *Atofp4* mutant exhibited thicker interfascicular fiber cell wall and thinner xylary fiber cell wall (Li et al., 2011; Wang et al., 2011). AtOFP1 interacts with ATH1 to regulate flowering time and stem growth in *Arabidopsis* (Zhang et al., 2018). Besides, *AtOFP1* is involved in the development of male gamete and pollen activity as well as DNA repair (Hackbusch et al., 2005; Wang et al., 2010). In rice (*Oryza sativa*), overexpression of *OsOFP2* led to a series of variations in plant height, leaf morphology, seed shape and abnormity of vascular bundles in stems; *OsOFP2* suppresses the expression of *GA20ox* by modulating the function of KNOX-BELL and inhibits lignin biosynthesis, thereby affecting vasculature development (Schmitz et al., 2015). In radish (*Raphanus sativus*), *RsOFP2.3* is negatively associated with tuberous root elongation and the tuberous root shape (Wang et al., 2020). In peach (*Prunus persica*), *PpOFP1* regulates fruit shape (Cirilli and Rossini, 2021), and another similar study showed that PpOFP1 physically interacts with a ZF-HD_dimer domain protein PpZFHD1 and regulates the salt tolerance of tomato (Tan et al., 2021). In cotton (*Gossypium hirsutum*), *GhOFP4* was found to regulate fiber development (Gong et al., 2014). In *Capsicum annuum*, *CaOFP1* is involved in fruit shaping, and its different expression profiles would result in different shapes *via* negatively affecting the expression of *CaGA20ox1* (Tsaballa et al., 2011), and gene silencing of *CaOFP20* increased the fruit length (Borovsky et al., 2022). Ectopic expression of *CsOFP12-16c* from cucumber (*Cucumis sativus*) in *Arabidopsis* affects the silique development and causes blunt and shorter siliques (Han et al., 2022). Previous studies have also suggested that *CmOFP13* may control the fruit shape in melon (*Cucumis melo*) (Monforte et al., 2014; Ma et al., 2021). Also, the expression of *MaOFP1* was reported to be negatively associated with fruit ripening in banana (*Musa paradisiaca*) (Liu et al., 2015b). Although more and more functions of OFPs have been reported in many crops, there are still numerous unknown features remaining to be discovered.

*B. napus* (2n = 38, AACC) is a polyphyletic polyploidy formed by *B. oleracea* and *B. rapa* (Nagaharu, 1935; Allender and King, 2010), and has experienced whole genome duplication (WGD) (Jiao et al., 2011; Chalhoub et al., 2014). *Brassica napus napus* still retains two sets of chromosomes corresponding to *B. oleracea* and *B. rapa* (Chalhoub et al., 2014). Hence, there are many duplicate genes from two sub-genomes or even the earlier progenitor *Arabidopsis*. Generally, *Arabidopsis* can serve as an efficient model plant in functional gene research. *AtOFPs* have been found to have different functions. However, multiple copies of *OFP* genes have been rarely reported in its closely related species *B. napus*. In this study, we identified the *BnOFP* gene family at the genomic level, analyzed its evolution from *Arabidopsis* to *B. napus* and compared the difference between copies. A new locus *BnOFP13_2* significantly correlated with NSPS was identified, which can be considered as a novel function of *OFPs*. RNA profiling during silique development suggested that *BnOFP13_2* negatively regulates NSPS. Our findings provide evidence for the functional diversity of OFP gene family and important implications for oilseed rape breeding.

## Results

### Identification and chromosomal distribution of ovate family proteins gene family in *Brassica napus*

**FIGURE 1:**
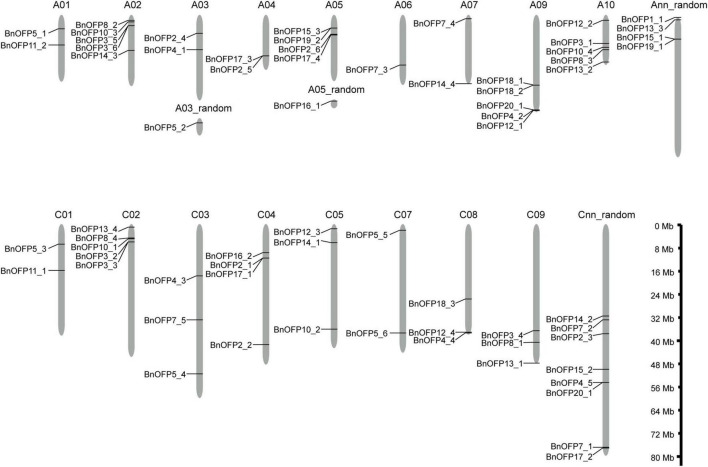
Chromosomal locations of *BnOFPs*. The 67 *BnOFP* genes were located on different chromosomes of two sub-genomes. Random chromosome means that the sequence was mapped to corresponding chromosome but the physical location was unknown. Length of chromosomes is also shown in the figure.

By using the reported 20 AtOFP or AtOFP-like protein sequences as queries, a total of 67 *OFP* genes were identified through BLAST in *B. napus* and *Arabidopsis* databases, which were renamed according to their orthologous genes in *Arabidopsis*, and their physical and chemical properties were analyzed (Supplementary Table 1). Among the 20 *AtOFP* genes, *AtOFP6* and *AtOFP9* had no orthologous gene in *B. napus*; *AtOFP1* had only one orthologous gene *BnOFP1_1*; while *AtOFP2*, *AtOFP3* and *AtOFP5* all had six orthologous genes. The other *13 AtOFP* genes, respectively, had two to five orthologous genes in *B. napus* (Supplementary Table 1). The 67 *BnOFP* genes were unevenly distributed on the 20 chromosomes of A sub-genome (34 *BnOFP* genes) and C sub-genome (33 *BnOFP* genes) (Figure 1). In the A sub-genome, A08 chromosome had no *BnOFP* gene; chromosome A06 contained one *BnOFP* gene; and A02, A09, and A10 chromosomes, respectively, harbored five *BnOFP* genes. On the same chromosome, some *BnOFP* genes were closely located, while some other genes were far away from each other. For instance, *BnOFP17_3* and *BnOFP2_5* were closely located on chromosome A04, while *BnOFP7_4* and *BnOFP14_4* were located on both ends of chromosome A07. In the C sub-genome, chromosome C06 contained no *BnOFP* member, and eight *BnOFP* members were located on unknown chromosomes, while other chromosomes, respectively, harbored two to five members (Figure 1). These results suggested the occurrence of genome rearrangement and gene loss during polyploidization.

### Phylogenetic and syntenic relationship of ovate family proteins gene family in *Brassica napus*

**FIGURE 2:**
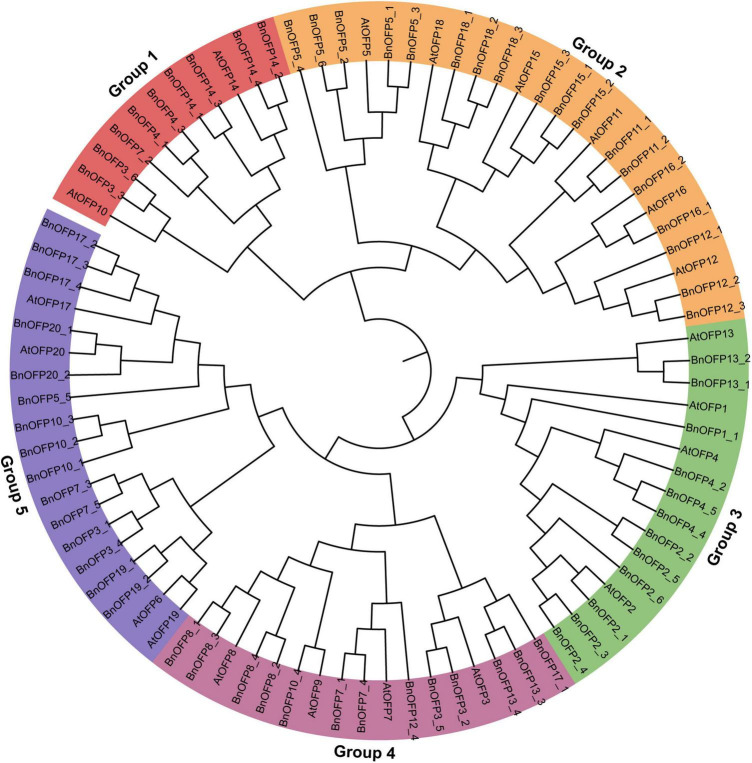
Phylogenetic relationship analysis of ovate family proteins (OFPs) in *Arabidopsis* and *Brassica napus*. The 20 *AtOFP* genes and 67 *BnOFP* genes were clustered into five groups. Members of each group were represented by different background color.

Phylogenetic relationship is an important indicator for gene functional research. The 87 OFP proteins from *B. napus* and *Arabidopsis* were used to construct a phylogenetic tree. As a result, five groups were clustered based on the sequence alignment (Figure 2 and Supplementary Table 2). Group 2 was the largest clade (24 proteins), followed by group 5 (19 proteins), group 4 (17 proteins), group 3 (16 proteins), and group 1 (11 proteins). Among the five groups, group 2 and group 3 contained some *AtOFPs* and all their corresponding *B. napus* orthologous genes. For example, group 2 included *AtOFP5*, *AtOFP11*, *AtOFP12*, *AtOFP15*, *AtOFP16*, *AtOFP18* and *BnOFP5*, *BnOFP11*, *BnOFP12*, *BnOFP15*, *BnOFP16*, and *BnOFP18*. On the contrary, some *AtOFPs* and their orthologous *BnOFPs* were not clustered together. For example, *AtOFP10* was clustered in group 1, but the *BnOFP10* orthologs were clustered in group 4 or group 5. Genes with similar functions tended to be clustered in the same group. For instance, *AtOFP1* and *AtOFP4* were both involved in secondary cell wall formation and thus clustered in group 3 (Li et al., 2011; Wang et al., 2011); *AtOFP17* and *AtOFP20* were both paralogous genes from segmental duplication blocks, and were also clustered in the same group (Liu et al., 2014; Figure 2). During the polyploidization from *Arabidopsis* to *B. napus*, members in different groups had undergone loss-of-function and function divergence. Therefore, the *BnOFP* gene family members may participate in different biological processes in plant development.

**FIGURE 3:**
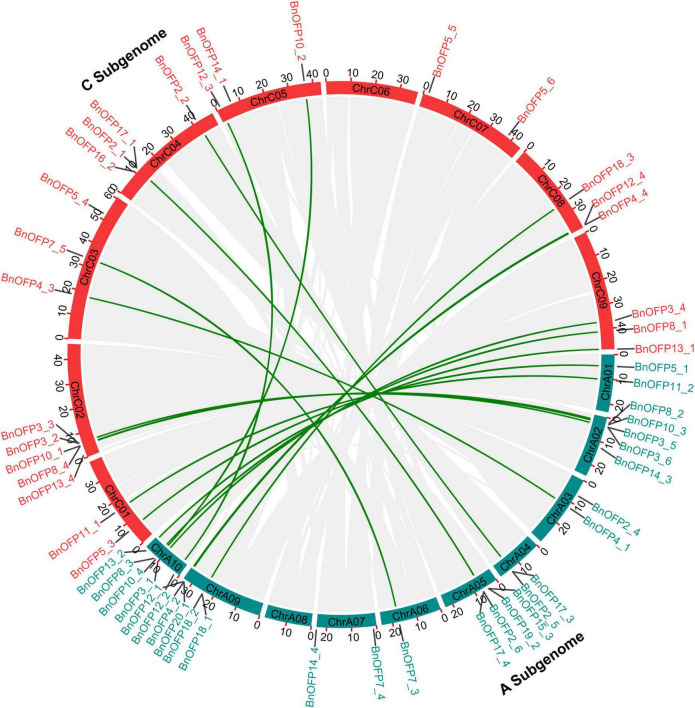
Syntenic relationship between *Brassica napus* A and C sub-genome. Chromosomes including *BnOFP* members of two sub-genomes are shown in different color. Gray lines in circle represent the syntenic genes or blocks, and dark green lines represent the syntenic genes in *BnOFPs*.

Since *B. napus* is of polyphyletic polyploidy with syntenic relationship between the sub-genomes, we analyzed the syntenic relationship between the *BnOFPs* in A and C sub-genomes (Figure 3). We analyzed the *53 BnOFPs* with specific chromosomal locations, and finally identified 19 pairs of syntenic genes. No syntenic gene was detected on chromosome A07 and A08 as well as on C06 and C07, while other chromosomes contained one to five syntenic genes. Chromosome A10 included the most syntenic genes (five genes), with two on chromosome C05 and three on C09. Pairs of syntenic genes were in the same subfamily, such as *BnOFP7_3* and *BnOFP7_5*, and *BnOFP2_2* and *BnOFP2_5* (Figure 3). Gene phylogenetic and syntenic relationship can be used to explore the functions of unknown genes. Hence, our results may be of great significance for gene functional research in *B. napus*.

### Gene structure and conserved domains of *BnOFP* genes

**FIGURE 4:**
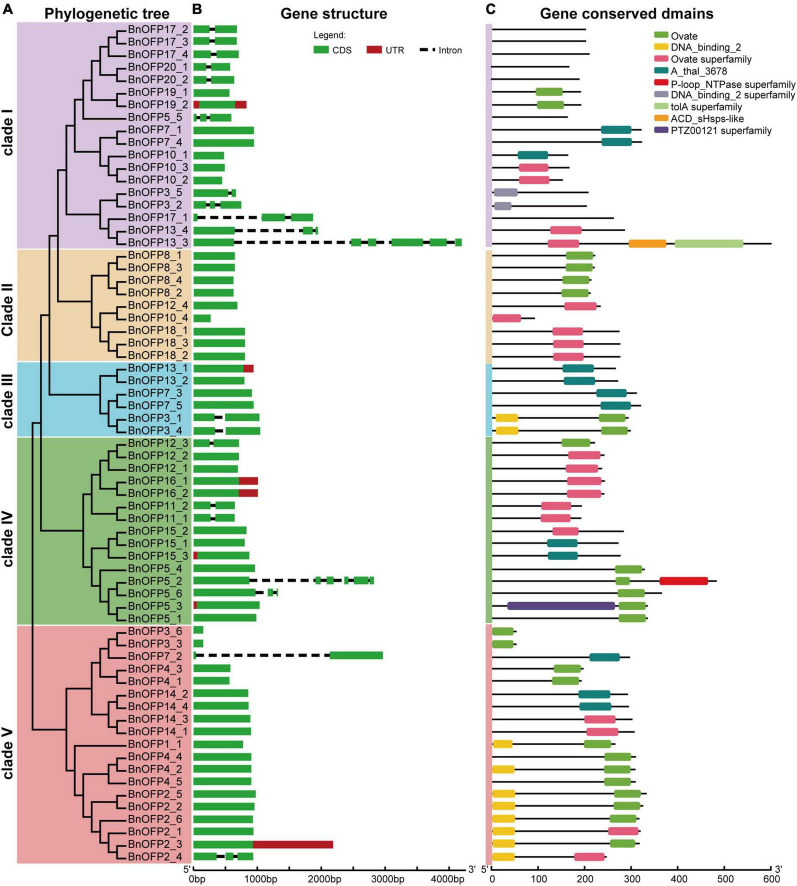
Gene structure and conserved domains of BnOFPs. **(A)** Phylogenetic tree of *BnOFP* genes. The 67 *BnOFP* genes were classified into five clades, each of which is illustrated with different colors. **(B)** Gene structure of the 67 *BnOFP* genes. Boxes represent the CDS and UTR section, and the lines indicate introns. **(C)** Gene conserved domains of the 67 *BnOFP* genes. Nine conserved domains are represented in different colored boxes, and the line means non-conserved sequence. Length scale is presented at the bottom.

Gene structure was analyzed to investigate whether there are differences among the 67 *BnOFP* genes. As expected, large differences were found in gene length and exon number among different members. The gene structure was displayed according to the five clades in the phylogenetic tree (Figure 4A). The gene length ranged from 153 to 4,195 base pairs (bp), and most genes were less than 1,000 bp. The exon number ranged from one to six, and 70.15% (47 out of 67) of the genes had only one exon (Figure 4B). Some genes displayed unique structures. For instance, only seven genes had untranslated region (UTR); *BnOFP15_3* and *BnOFP5_3* had upstream UTR, *BnOFP13_1*, *BnOFP16_1*, *BnOFP16_2*, and *BnOFP2_3* had downstream UTR; while *BnOFP19_2* had both upstream and downstream UTR (Figure 3B). This gene family had fewer introns, as well as great differences in intron length. *BnOFP17_1*, *BnOFP13_4*, *BnOFP13_3*, *BnOFP5_2*, and *BnOFP7_2* contained longer introns, and *BnOFP5_5* and *BnOFP3_2* had shorter introns. Some members derived from the same *Arabidopsis* gene showed the same gene length and structure, such as the *BnOFP8* and *BnOFP18* subfamily (Figure 4B).

We also analyzed the gene conserved domains, and identified a total of nine conserved domains. Ovate and ovate superfamily domain were detected in 68.66% of the *BnOFP* members (Figure 4C). Most members contained one domain, and a few members harbored two or three domains. Some domains were rarely detected, such as the P-loop_NTPase superfamily and PTZ00121 superfamily domains, which were only detected in *BnOFP5_2* and *BnOFP5_3*, respectively. However, 31.34% members had no ovate family domains, and seven members in clade I had no conserved domain. This loss of domain may be caused by genome polyploidization. These results may greatly help the research on the functional conservation and divergence of *BnOPFs* in *B. napus* evolution.

### Differential expression of *BnOFPs* in different tissues

**FIGURE 5:**
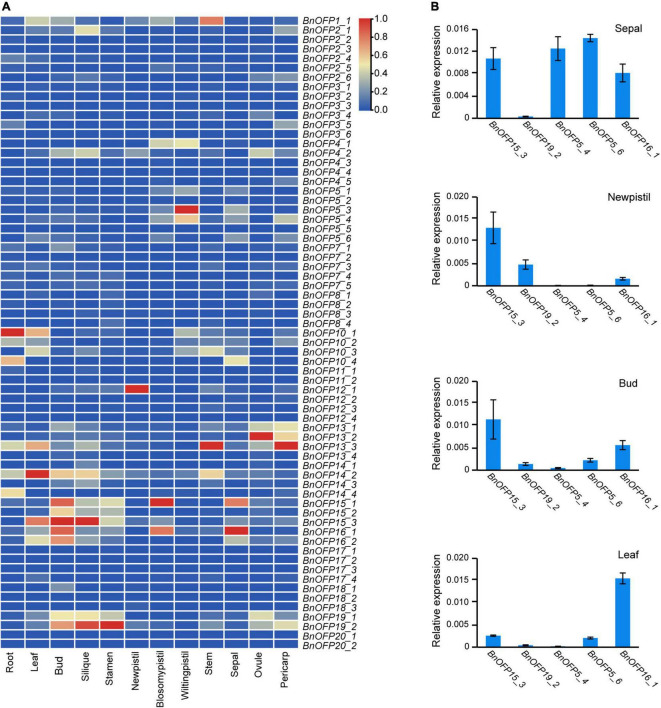
Expression patterns of *BnOFP* genes. **(A)** Heatmap of gene expression patterns. Expression levels of 67 *BnOFP* genes in twelve tissues of ‘ZS11’ obtained from FPKM of transcriptome analysis. **(B)** Verification of the expression level by qRT-PCR. Five genes and four tissues were used to verify the expression level in transcriptome analysis by qRT-PCR.

With the rapid development of RNA sequencing technology, transcriptome analysis has been widely used in gene functional research. The expression level of a gene is related to its functional performance. The FPKM (fragments per kilobase of exon model per million mapped fragments) value from RNA sequencing can effectively represent the gene expression levels and be used to analyze the biological processes in plant tissues in different stages and environments. In this study, a transcriptome analysis of twelve tissues in *B. napus* cultivar line ‘ZS11’ was performed to analyze the expression pattern of *BnOFPs*. We found that the expression of 67 *BnOFPs* varied greatly among the twelve tissues (Figure 5A and Supplementary Table 3). The majority of the genes had generally low expression (Supplementary Table 3). *BnOFP5_1*, *BnOFP5_6*, *BnOFP10_2*, *BnOFP10_4*, *BnOFP12_2*, and *BnOFP12_3* had relatively higher expression in the sepal, while *BnOFP14_2*, *BnOFP16_2*, *BnOFP7_1*, *BnOFP18_1*, *BnOFP14_3*, *BnOFP15_2*, and *BnOFP15_3* exhibited relatively higher expression in the bud. *BnOFPs* were lowly expressed in most tissues. For example, all the genes had low expression in the stem, while in the leaf and silique, only *BnOFP17_7* and *BnOFP2_1* had relatively high expression (Figure 5A). Ten genes showed no expression in any tissue, probably because they have lost their functions during evolution. A qRT-PCR experiment was then performed to verify the transcriptome data, and the results of five genes in four tissues are presented in Figure 5B. The expression of *BnOFP5_4*, *BnOFP5_6*, and *BnOFP16_1* was obviously higher in the sepal and lower in the new pistil, while that of *BnOFP19_2*, *BnOFP5_4*, and *BnOFP5_6* was obviously low in the bud and stem (Figure 5B). The qRT-PCR experimental results were consistent with the transcriptome results, verifying the reliability of the transcriptome data. The expression pattern of *BnOFPs* suggested the occurrence of functional divergence in this gene family of *B. napus* during evolution.

### Association of *BnOFP13_2* with number of seeds per silique

**FIGURE 6:**
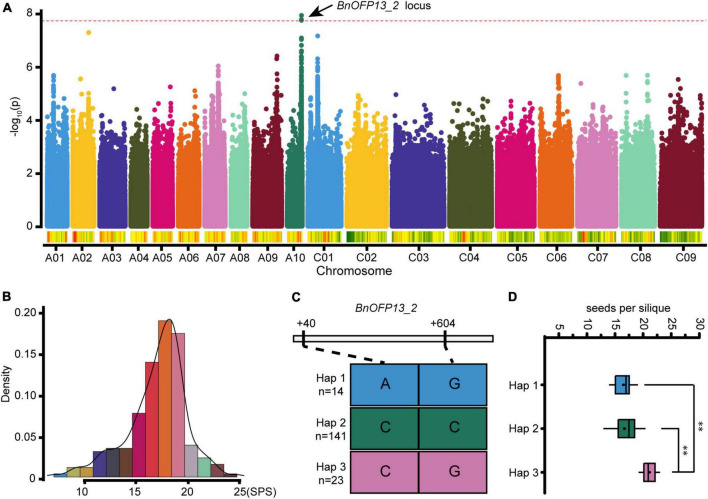
Genome wide association study (GWAS) and haplotype analysis. **(A)** Manhattan map of GWAS. The red dotted line represents the threshold value 7.72. Dots above the red dotted line indicate the significant loci associated with NSPS. **(B)** Histogram of frequency distribution. Statistical analysis of NSPS phenotype used in GWAS. Phenotype variations of 204 accessions are presented. **(C)** Haplotype analysis of *BnOFP13_2*. All the accessions were classified into three haplotypes based on the two non-synonymous SNPs of *BnOFP13_2*. **(D)** Haplotype analysis. Statistical analysis of accessions in each haplotype, and the phenotype value shows significant differences. **p < 0.01 in ANOVA test.

Genome wide association study is a new approach to precisely predict the corresponding genes or QTLs involved in the regulation of complex traits of plants based on linkage disequilibrium (LD) (Nordborg and Weigel, 2008). In this study, GWAS for NSPS was performed with a natural population consisting of 204 accessions. The phenotype value ranged from 7.06 to 24.68, exhibiting large differences among different accessions (Figure 6B). Finally, a significant locus was identified on chromosome A10. The 16.39–16.47 Mb block on A10 contained a *BnOFP* member, and the most significant SNP was located around *BnOFP13_2* (Figure 6A and Supplementary Table 4). Therefore, we speculated that *BnOFP13_2* might be associated with NSPS in *B. napus*, and then analyzed the SNP of *BnOFP13_2* in the 204 accessions. Two homozygous non-synonymous SNPs were obtained at the position of +40 and +604 bp of gene sequence. There were three classified haplotypes based on the two SNPs. Haplotype 1 comprised 14 accessions, and haplotype 2 and haplotype 3, respectively, had 141 and 23 accessions (Figure 6C). The accessions in haplotype 1, haplotype 2, and haplotype 3 had the average phenotype value of 16.50, 16.67, and 21.11, respectively, indicating that haplotype 3 had significantly higher NSPS than haplotype 1 and haplotype 2 (Figure 6D). These results suggested that *BnOFP13_2* is associated with NSPS.

### Negative regulation of *BnOFP13_2* on number of seeds per silique in early silique development

**FIGURE 7:**
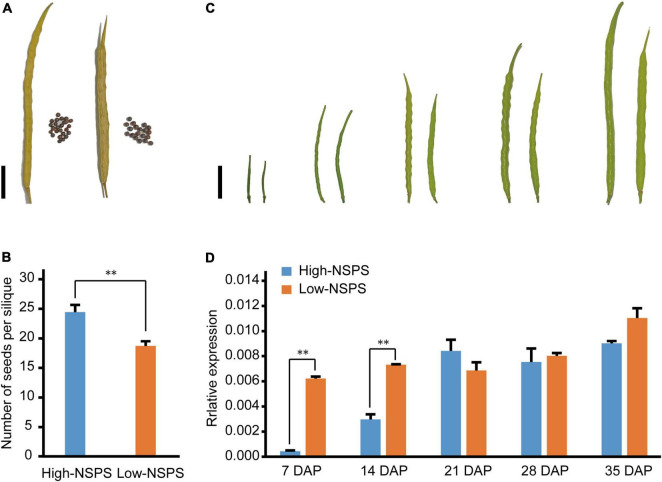
Phenotype of siliques at different developmental stages and expression levels of BnOFP13_2 in high-NSPS variety and low-NSPS variety. **(A)** Mature silique of high-NSPS variety (left) and low-NSPS variety (right), bar: 2 cm. **(B)** Statistical analysis of NSPS in high-NSPS variety and low-NSPS variety. **(C)** Phenotype of high-NSPS variety and low-NSPS variety siliques at 7, 14, 21, 28, and 35 DAP, bar: 2 cm. **(D)** Relative expression levels of *BnOFP13_2* in high-NSPS variety and low-NSPS variety silique at different developmental stages. ^**^p < 0.01.

To investigate how *BnOFP13_2* affects NSPS in *B. napus*, we detected the RNA profiles of *BnOFP13_2* at different silique developmental stages in two varieties, respectively, with high NSPS (‘ZC520’) and low NSPS (‘ZC519’). ‘ZC520’ averagely had 24 seeds per silique and ‘ZC519’ only had 18 seeds per silique (Figures 7A,B). The siliques at 7 days after pollination (7 DAP), 14, 21, 28, and 35 DAP were collected to analyze the dynamics of the expression level of *BnOFP13_2* (Figure 7C). At 7 and 14 DAP, the expression of *BnOFP13_2* was significantly higher in ‘ZC520’ than in ‘ZC519’ (Figure 7D). From 21 to 35 DAP, the expression level of *BnOFP13_2* was constantly high without significant difference between the two varieties (Figure 7D). In ‘ZC520’ (high-NSPS variety), the expression of *BnOFP13_2* was low in the early developmental stage of silique, which then increased slowly until 21 DAP and remained constant until maturity; while in ‘ZC519’ (low-NSPS variety), *BnOFP13_2* showed a high expression level at almost all developmental stages (Figure 7D). These results suggested that the accumulation of *BnOFP13_2* transcripts at the early developmental stage of silique is negatively correlated with NSPS. Therefore, we speculated that *BnOFP13_2* suppresses the formation or development of ovule, which is similar to the previous finding in *Arabidopsis*, but the specific molecular mechanism remains to be further studied.

## Discussion

**FIGURE 8:**
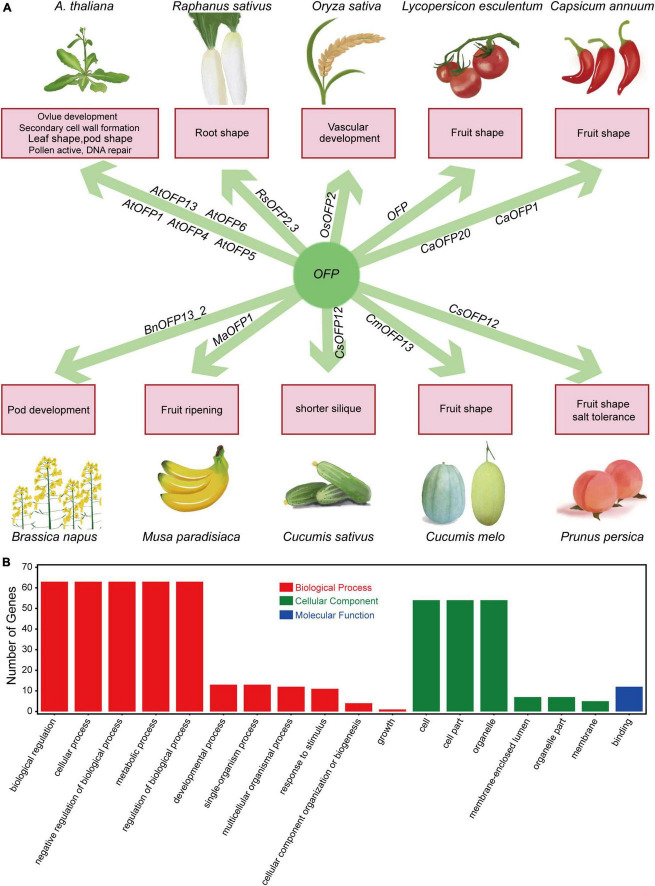
Brief summary of *OFP* functions and GO annotations of the 67 *BnOFP* genes. **(A)** Function summary of *OFPs* in common crops. The crop pictures are painted. **(B)** GO annotations of *OFP* genes in *B. napus*. Gene numbers and annotations in biological processes, cellular components and molecular functions are shown in the figure.

More than 20 years ago, OVATE was a well-known QTL for its effect in controlling the pear shape of tomato fruit, which was then mapped and cloned on chromosome 2 (Ku et al., 2001; Liu et al., 2002). The protein and RNA profiles have suggested that OVATE is a novel class of proteins named as OFPs (ovate family proteins) (Liu et al., 2002). Owing to their effects on fruits, *OFPs* have been studied in many fruit crops, and the results have indicated that *OFPs* have great influence on a variety of aspects in plants, including fruit shape (Liu et al., 2002; Tsaballa et al., 2011; Cirilli and Rossini, 2021; Han et al., 2022), tuberous root shape (Wang et al., 2020), ovule development (Pagnussat et al., 2007), secondary cell wall formation (Li et al., 2011; Wang et al., 2011), vasculature development (Schmitz et al., 2015), fruit ripening (Liu et al., 2015b), DNA repair (Wang et al., 2010), floral organs, compound leaf and silique (Liu et al., 2002; Wang et al., 2011). Here, we briefly summarized the functions of OFPs in *Arabidopsis* and some common crops (Figure 8A), and we believe that more functions of OFPs will be gradually detected in plants. There has been no report about the functions of *OFPs* in *B. napus*. In this study, 67 *BnOFP* members were checked with gene ontology (GO) annotations (Figure 8B). As a result, 63 members were involved in biological regulation, cellular process, metabolic process, negative regulation of biological process and regulation of biological process; 52 members were related to cell part and organelle component; and 12 members had the binding molecular function (Figure 8B). These results suggested that *BnOFPs* play different roles in plant development. We further identified and characterized the *OFPs* in *B. napus*, as well as analyzed their phylogenetic relationship with the orthologs in *Arabidopsis* and the syntenic relationship between the two sub-genomes. We also analyzed the gene structure, conserved domain and expression pattern, and the differences in the gene structure and expression could provide a theoretical basis for functional research of this gene family. In addition, a novel function of *OFPs* was identified through GWAS, and haplotype analysis suggested that *BnOFP13_2* is significantly associated with NSPS. Further expression profiling at the silique developmental stage indicated that *BnOFP13_2* negatively regulates NSPS during early silique development. Our results may provide important information for rapeseed breeding.

In *Arabidopsis*, *AtOFP1* was firstly identified as a transcriptional repressor of cell elongation in many organs. An increase in the expression of *AtOFP1* reduced the length of hypocotyl, inflorescence stem, cauline leaf, rosette leaf, floral organ, and silique (Wang et al., 2007). Overexpression of *AtOFP2*, *AtOFP4*, and *AtOFP7* led to similar phenotypes to overexpression of *AtOFP1* such as round and curled leaves, implying that these three genes are also transcriptional repressors of cell elongation (Wang et al., 2007; Li et al., 2011). Overexpression of *AtOFP6* and *AtOFP8* resulted in thick and cyan rosette leaves in plants, while that of *AtOFP13*, *AtOFP15*, and *AtOFP16* led to short and blunt-ended siliques (Wang et al., 2011). In this study, high expression of *BnOFP13_2* was detected at early developmental stage of siliques in low-NSPS variety. Therefore, it can be speculated that *BnOFP13_2* negatively regulates NSPS by reducing silique length and inhibiting ovule development at early developmental stage.

Since *OFPs* are negative regulators of plant growth and development, the underlying mechanisms have received great attention. Although the mechanisms remain largely unknown, there has been some evidence implying that *OFPs* may directly regulate the expression of the target genes. In *Arabidopsis*, AtOFP1 contains a putative NLS domain and is located in the nucleus, and a yeast one-hybrid experiment indicated that *AtOFP1* represses the expression of the reporter gene. These results suggest that *AtOFP1* is a transcription repressor directly regulating gene expression (Wang et al., 2007, 2011). In addition, *AtOFP1* also contains the LxLxL domain, which was also found in ERF transcription factors and is necessary for transcriptional repression (Hiratsu et al., 2003). On the other hand, *OFPs* were found to form a complex to perform their functions *via* interacting with other proteins. For instance, AtOFP4 and AtOFP1 interact with KNAT7 and BLH6 to regulate secondary cell wall formation (Li et al., 2011); AtOFP1 coordinates with ATH1 to affect flowering time and stem growth (Zhang et al., 2018); AtOFP1 has interaction with BLH3 to regulate the transition from vegetative phase to reproductive phase (Zhang et al., 2016); AtOFP5 cooperates with KANT2 and BLH1 in regulating female gametophyte development (Pagnussat et al., 2007); MaOFP1 interacts with MuMADS1 to regulate fruit ripening (Liu et al., 2015b); GhOFP4 coordinates with GhKNL1 in mediating secondary cell wall formation (Gong et al., 2014); and OsOFP2 interacts with OsKNAT7 and BLH6-like1 to mediate vascular development (Schmitz et al., 2015). With a better understanding of these negative regulators, *OFPs* can be better used for crop improvement *via* the gene editing technology.

In plants, polyploidization occurs at high frequency. It has been demonstrated that about 25–30% of the existing flowering plants are polyploids and have not been diploidized yet (Wood et al., 2009; Scarpino et al., 2014). Polyploids are generally classified into autopolyploids and allopolyploids, with the former being formed through doubling of one chromosome set, while the latter being formed *via* hybridization or merging of different chromosome sets in different species (Ramsey and Schemske, 1998; Barker et al., 2016; Van de Peer et al., 2017). Allopolyploidy is involved in epigenetic remodeling and changes in gene expression, which will contribute to a higher adaptive potential (Yoo et al., 2014; Hu et al., 2015b; Van de Peer et al., 2017). It is widely accepted that polyploids have increased the mutational robustness and adaptability compared with diploids. Polyploids can overcome sympatrically speciate and cytotype minority exclusion for rapid adaptation, which is also owing to their higher genomic plasticity than diploids (te Beest et al., 2012; Schoenfelder and Fox, 2015). Gene duplication generally has four modes, including the tetraploid, segmental, tandem, and transpositional modes, with each mode retaining genes in a biased method (Neufeld et al., 1991; Freeling, 2009). After genome or segment duplication, some duplicated genes are often lost (Freeling, 2009). Polyploidization or WGD is an important driving force for evolution in animals and plants (Abdel-haleem, 2007; Edger and Pires, 2009), and the success of angiosperms is partially attributed to WGD (De Bodt et al., 2005). In *Arabidopsis*, *OFPs* are involved in different functions. After polyploidization into *B. napus*, the *OFP* members have experienced gene doubling, loss and functional divergence, which may lead to the appearance of novel gene structure, conserved domains and functions. In this study, seven *BnOFP* members were detected to have no ovate or related domains (Figure 4C), which might have been caused by loss-of-function during polyploidization.

## Conclusion

In this study, we identified 67 *BnOFP* genes at the genome level, analyzed their evolution from *Arabidopsis* to *B. napus* and compared the gene structure and conserved domains between copies. We also identified a new potential locus significantly correlated with NSPS, which is a novel function of *OFPs*. RNA profiling in different stages of silique development suggested that *BnOFP13_2* negatively regulates the NSPS by decreasing the silique length and inhibiting the ovule development at early developmental stage. Our findings provide evidence for the functional diversity of *OFP* gene family and new implications for oilseed rape breeding.

## Materials and methods

### Plant materials and phenotype identification

The 204 natural accessions and ‘ZS11’ used for GWAS analysis and qRT-PCR experiment were obtained from the Key Laboratory of Biology and Genetic Improvement of Oil Crops at OCRI and planted in our experimental field in WuHan, HuBei province, China. These materials used for GWAS were collected from all over the world, including 52 spring accessions, 40 winter accessions and 112 semi-winter accessions, and were cultivated under natural growing conditions. The phenotype data were collected in 1 year (2017–2018). Planting was conducted with twelve plants in a row and a row spacing of 30 cm. All varieties were self-bred for many generations and were pure inbred lines. Ten siliques from different parts of the main inflorescence were collected to evaluate the NSPS. The phenotype of each variety was represented by the average of three biological replicates.

### Identification of *BnOFPs* in *Brassica napus*

The *OFP* gene protein sequences of *Arabidopsis* were obtained from the TAIR database^1^, and used to search for the *OFP* genes in *B. napus*. The annotation and genome information of *B. napus* cultivar ‘Darmor-bzh’ and corresponding orthologous genes in *Arabidopsis* were obtained from the BRAD (Brassicaceae Database) database^2^ (Chalhoub et al., 2014). The HMMER3.0^3^ was used to search for OFP genes in *B. napus* (E value was set to 1e-5).

### Gene structure and conserved domain analysis

Gene structure files of *BnOFPs* were downloaded from EnsemblPlants database^4^, and illustrated through GSDS_2.0_ (Gene Structure Display Server) online tools^5^ (Hu et al., 2015a). Gene conserved domains were analyzed through CD-search in NCBI (National Center for Biotechnology Information) database^6^ using the amino acid sequence, and illustrated through TBtools (Chen et al., 2020).

### Phylogenetic and syntenic relationship analysis

The protein sequences of *OFPs* in *Arabidopsis* and *B. napus* were used to construct a phylogenetic tree using the MEGA software. The beautification of phylogenetic tree was conducted with iTOL online tools.^7^ The syntenic relationship of gene or block between two sub-genomes were obtained from the BRAD database (Chalhoub et al., 2014). The syntenic relationship figure was drawn through TBtools (Chen et al., 2020).

### Transcriptome analysis and qRT-PCR analysis

The transcriptome data of twelve tissues in ‘ZS11’ used for the expression pattern analysis were obtained from our lab and have been already published (SRA accession: PRJNA474576) (Li et al., 2019b). The heatmap was drawn through TBtools (Chen et al., 2020). The sepal, leaf, bud, and newpistil samples used for RNA extraction were collected in experimental field. The silique samples were collected at 7, 14, 21, 28, and 35 DAP. All the samples had three biological replicates. The TRIzol reagent (Invitrogen, Carlsbad, CA, United States) was used for total RNA extraction. About 2 μg RNA was used to reverse transcribe using the PrimeScript™ RT reagent Kit (TaKaRa Co., Ltd., Beijing, China). The *B. napus* gene *BnaA10g22340D* was used as the reference gene. The relative expression was evaluated using the 2^–ΔΔCt^ method (Livak and Schmittgen, 2001).

### Genome wide association study analysis in natural population

The genomic DNA of all plants was extracted from tender rosette leaves using the modified CTAB method (Allen et al., 2006). The SNP data were obtained from 7 × re-sequencing data mapped to the reference genome ‘Darmor-bzh’. Re-sequencing was performed by the commercial Illumina HiSeq XTen service (BGI-Shenzhen, China). The SNPs were finalized under the minor allele frequency <0.05, and 2611513 valid SNPs were obtained at last, the original SNPs were obtained from published data of our lab (Ding et al., 2020). GWAS for NSPS was performed with the R package using the general linear model (Yin et al., 2021). The threshold value was set to *p* < -log_10_(0.05/*N*), where *N* represents the number of used SNPs.

## Data availability statement

The datasets presented in this study can be found in online repositories. The names of the repository/repositories and accession number(s) can be found in the article/Supplementary material.

## Author contributions

JL, XhC, and JH designed the research. SL supervised the research. JL and YW performed the experiments and wrote the manuscript. YW, XbC, and XZ collected the data. MX helped analyze the data. LL and YL provided the plant materials. XhC and JH revised the manuscript. All authors contributed to the article and approved the submitted version.

## Footnotes

1
https://www.arabidopsis.org/

2
http://brassicadb.cn/#/

3
http://www.hmmer.org/

4
http://plants.ensembl.org/index.html

5
http://gsds.gao-lab.org/

6
https://www.ncbi.nlm.nih.gov/

7
https://itol.embl.de/
